# Oral health status and the epidemiologic paradox within latino immigrant groups

**DOI:** 10.1186/1472-6831-12-39

**Published:** 2012-09-07

**Authors:** Vladimir W Spolsky, Marvin Marcus, Claudia Der-Martirosian, Ian D Coulter, Carl A Maida

**Affiliations:** 1 Division of Public Health and Community Dentistry. School of Dentistry, University of California, Los Angeles, U.S.A; 2 Division of Oral Biology & Medicine, School of Dentistry, University of California, Los Angeles, U.S.A; 3 RAND Corporation, Santa Monica, CA, U.S.A

**Keywords:** Oral health status index, Immigrant, Latinos, Epidemiologic paradox

## Abstract

**Background:**

According to the United States census, there are 28 categories that define “Hispanic/Latinos.” This paper compares differences in oral health status between Mexican immigrants and other Latino immigrant groups.

**Methods:**

Derived from a community-based sample (N = 240) in Los Angeles, this cross-sectional study uses an interview covering demographic and behavioral measures, and an intraoral examination using NIDCR epidemiologic criteria. Descriptive, bivariate analysis, and multiple regression analysis were conducted to examine the determinants that are associated with the Oral Health Status Index (OHSI).

**Results:**

Mexican immigrants had a significantly higher OHSI (p < .05) compared to other Latinos. The multilinear regression showed that both age and gender (p < .05), percentage of untreated decayed teeth (p < .001), number of replaced missing teeth (p < .001), and attachment loss (p < .001) were significant.

**Conclusions:**

Compared with the other Latino immigrants in our sample, Mexican immigrants have significantly better oral health status. This confirms the epidemiologic paradox previously found in comparisons of Mexicans with whites and African Americans. In this case of oral health status the paradox also occurs between Mexicans and other Latinos. Therefore, when conducting oral health studies of Latinos, more consideration needs to be given to differences within Latino subgroups, such as their country of origin and their unique ethnic and cultural characteristics.

## Background

An appreciation for the diversity between ethnic groups within the United States has emerged in the dental literature, especially with respect to health and health care needs [1]. The idea of diversity among ethnic groups is even newer and is particularly important when considering Latinos, where the diversity among those covered by the term, “Latinos,” is extensive. The lack of data on Latino sub-populations was the impetus to establishing the national Hispanic Health Research Consortium in 1986. In reviewing twenty-one data collection agencies of the Department of Health and Human Services, Delgado and Estrada [2] found only three that collected data on Mexican Americans and only one that collected data on Puerto Ricans, Cuban Americans and Central-South Americans. Latinos represent the fastest growing minority population in the United States. It is imperative that more attention be paid to the heterogeneity of Latino sub-populations in future studies [3,4].

Among Latinos, Mexican Americans constitute the largest subgroup at the national, state and local levels [5]. In cities, like Los Angeles, there are many Latino subgroups whose numbers are modest when compared to Mexican-Americans. According to the 2010 U.S. Census, there were 4.7 million “Hispanics” in Los Angeles County, California, which include Mexican-born and U.S.-born Mexicans, Central and South Americans [6]. Los Angeles has become a “social laboratory” for understanding ethnic heterogeneity in health care. The 2010 Census describes the Latino population by origin and includes Cubans, Mexicans, Puerto Ricans, South or Central Americans and other Spanish persons under the collective term of “Hispanic/Latinos” [6].

**Figure 1:**
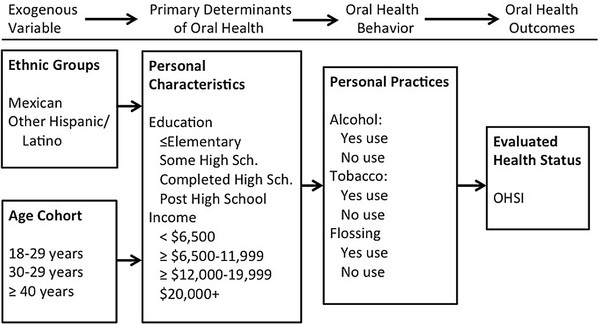
Conceptual Model of Ethnicity and Oral Health Outcomes.

Ethnicity has been identified in models of health behavior and health services utilization as a significant variable. For example, in Andersen and Davidson’s Behavioral Model (Figure 1) [7], ethnicity, like other *exogenous* variables, such as age and gender*,* affects the *primary determinants of oral health*. Within the original version of the model, a patient’s personal characteristics, the dental care system, and features of the external environment, together impact key oral health outcomes. The Behavioral Model has been adapted for this study as a framework to guide the research in determining how ethnicity and other socio-demographic factors can affect oral health status. 

The Oral Health Status Index (OHSI) is used in this research as a quantitative means of measuring evaluated oral health status outcomes [8]. It integrates the status of the teeth and periodontium into one numerical score. The OHSI was used by Marcus *et al.* (1983) [8] to compare low income populations in Los Angeles and New York City and found that the OHSI scores were significantly higher in Los Angeles than in New York. Deporter *et al.* (1988) [9] used the OHSI to compare capitation enrollees with those enrolled in voluntary dental plans. Manninen *et al.* (1994) [10] used the OHSI to compare skilled workers enrolled in either fee-for-service or capitation plans and found no difference between these two groups. Lang *et al.* (1997) [11] utilized the OHSI to compare African-Americans and whites in Michigan and found the index to be an effective measure of clinical oral health status and quantified the disparities in demographic and other variables. Spolsky *et al.* (2000) [12] established the validity of the OHSI using Latino populations. Otsuru *et al.* (2006) [13] used the OHSI as an outcome measure to examine the difference between native Japanese and migrant workers and found that the OHSI scores of native Japanese were significantly higher than those of the migrant workers.

Markides and Coreil [14] described the general health status of Mexicans relative to African Americans and whites, documenting the epidemiologic paradox in terms of a number of clinically determined health conditions and also self-perceived health. Other studies have confirmed the epidemiologic paradox for low birth weight [15] and infant mortally [16]. Apparently, clinical oral health may also demonstrate the epidemiologic paradox whereby Latinos born in Mexico have better oral health status than those born in other Latino countries despite low utilization and less access to dental care. According to Markides and Coreil [14], possible explanations for this difference may be related to selected migration, whereby Mexicans are more likely to be from rural areas while other Latinos are more likely to come from urban areas. This may be reflected in oral health practices and diet. Extended family support is another factor identified by Markides and Coreil and is a more subtle issue since Latino cultures tend to have strong family ties. In this paper we examine the epidemiologic paradox by comparing differences in oral health status within Latinos, specifically between Mexican immigrants and other Latino immigrant groups.

## Methods

An adaptation of the Behavioral Model provides the conceptual approach for this study. The exogenous variables include ethnicity and age; while personal characteristics include education and income; and oral health behavior include both positive (flossing) and negative health behaviors (alcoholic consumption and tobacco use)**.**

### Study sample

A community-based sample of 240 low-income immigrant Mexicans and other Latino immigrants being served by two UCLA community dental clinics was recruited in Venice, California from January-September 1993 to provide insight into the oral health status of Mexicans and other Latinos living in Venice, California (a section of the City of Los Angeles). Prior to this assessment, no other dental documentation existed concerning Latinos in Los Angeles. Venice has a population of over 31,000. Latinos comprise one-quarter of this population (7,750), and Mexicans comprise three-quarters of the total Latino population (5,813) [17]. The composition of this foreign-born sample was 157 Mexicans and 83 other Latinos. At the time of the study, Latino subgroups living in the Zip Code of the Venice Clinic had an average age of 36 years and incomes ≥ $12,000-$19,999. Over 50% of the participants in our sample had incomes ≤ $12,000. Even when this comparison is projected to the 2000 census data (no data was available from the 2010 census data), the participants in this study had lower incomes [18].

A flyer describing the study, in English and Spanish, was posted in waiting rooms of medical clinics serving this population in Venice as well as the two participating dental clinics. All participants signed an English or Spanish language consent form approved by the Institutional Review Board of the University of California, Los Angeles before any clinical examination or interview took place. Participants were given a $5 honorarium if they completed an interview and a clinical dental examination. Although a few of the participants were scheduled as new patients, the vast majority were not clinic patients, but accompanied their children or other members of their family to the clinic. Of those in our sample who sought treatment, none received treatment prior to the administration of the interview and examination. Almost all persons who were asked to participate in the study agreed to do so. Of those who declined, the majority did so because of time constraints and their concerns about missing work. Only completely edentulous persons were excluded. The resultant sample compared favorably with the population of Latinos that reside in the Zip Codes contained in the Venice community; in terms of average age, however, their incomes were slightly lower.

### Interview

One interviewer who was bilingual and fluent in Spanish, and familiar with dental terms was trained and conducted face-to-face interviews with each subject. Interviews were conducted mostly in Spanish. Demographic questions included age, gender, income, education, ethnic classification, and country of birth. The behavioral questions covered measures on brushing, flossing, tobacco use, and alcohol use.

### Intraoral examination

Prior to the study, two dental examiners were standardized to the criteria and then calibrated using patients from the dental clinic. The senior of the two examiners served as the reference examiner. Duplicate examinations were conducted throughout the study to determine intra-examiner and inter-examiner reliability. The National Institute of Dental and Craniofacial Research (NIDCR) clinical criteria were used for examining tooth status and measures of periodontal destruction. [19]. Using weighted kappa values, intra-examiner reliability ranged from 0.7 to 0.9 and inter-examiner reliability from 0.5 to 0.8 depending on the specific index. The senior examiner performed approximately 80% of the examinations. All examinations were conducted in a dental operatory using current infection control methods and barrier techniques. Radiographs were not used during the clinical examinations.

### The oral health status index

The OHSI is an outcome measure that combines and weights the status of the teeth (frank decay, missing and replaced) and periodontium (i.e., specifically attachment level) into one numerical score. A more detailed description of the OHSI and its calculation is given in previous papers [8,20]. The five components of the OHSI are: Decayed Teeth (DT); Missing Teeth (MT); Free Ends, referring to the number of quadrants in the mouth in which all molars are clinically missing; Replaced Teeth (RT); and millimeters of Attachment Loss (AL) at the mesial facial surface that was subdivided into 4 to 6 mm of moderate AL and > 6 mm of severe AL. Scores are based on 32 teeth, and whole mouth scores per person.

### Statistical analysis

In order to determine the adequacy of the sample, a power calculation separated the Mexican immigrants from the other Latino immigrants. The power was calculated using mean OHSI scores as well as mean number of sound teeth. According to these calculations, 45 subjects in each group will yield 90% power to detect a difference of 4.60 OHSI units, between the Mexican immigrants and other Latino immigrant groups using a two-tailed 5% significance level. Therefore, there were sufficient numbers in our sample to determine significant differences**.** The descriptive analysis focused on the demographic and clinical measures (i.e., the Decayed, Missing, Filled permanent Teeth [DMFT] and periodontal disease measures). For the bivariate and multivariate analysis, the DMFT was transformed into percentage of decayed over decayed plus filled teeth, a measure of unmet needs for fillings; number of replaced teeth over missing teeth multiplied by 100 resulting in a ratio percentage that represents the degree of missing teeth that have been replaced. Chi-squared, paired t-tests and ANOVA were used to determine significant differences in the bivariate analysis. Multiple linear regression analysis, using the method of ordinary least squares, was used on epidemiologic measures with incremental addition of predisposing and enabling demographic and behavioral variables. The dependent variable in this analysis is the OHSI, and the independent variable is the place of birth, i.e., Mexico or other Latin American country. Covariates included epidemiologic, demographic and behavioral variables.

## Results

Table 1 describes the demographic and behavioral characteristics of the sample by number and percentage based on bivariate analysis. There were no significant differences in age between the Mexican and other Latino Immigrants. There were, however, significant differences by gender, education and income. None of the behavioral covariates of alcohol consumption, tobacco use and flossing showed significant differences. These bivariate findings do not provide us with the total picture because each set of variables does not take into account the interaction between all variables. In order to examine the influence of the other variables, a multiple linear regression analysis was conducted.

**Table 1:** Comparison of demographic and behavioral characteristics for Mexican and other Latino immigrants by Number and Percentage

**Variable**	**Total**		**Mexican Immigrants**		**Latino Immigrants**		**p-values**
	**n**	**%**	**n**	**%**	**n**	**%**	
**DEMOGRAPHIC**							
**Age**							
18–29 years	69	29%	44	28%	25	30%	
30–39 years	102	43%	71	45%	31	37%	
≥ 40 years	69	29%	42	27%	27	33%	ns
Total	240 ^*,†^	100%	157	100%	83	100%	
**Gender**							
Males	74	31%	39	25%	35	42%	
Females	166	69%	118	75%	48	58%	<.01
Total	240	100%	157	100%	83	100%	
**Education**							
≤ Elementary	125	52%	90	57%	35	42%	
Some High School or more	115	48%	67	43%	48	58%	<.05
Total	240	100%	157	100%	83	100%	
**Income**							
$0–$11,999	121	59%	72	57%	49	64%	
≥$12,000	83	41%	55	43%	28	36%	<.01
Total	204 ^‡^	100%	127	100%	77	100%	
**BEHAVIORAL**							
**Alcohol consumption**							
Never	156	65%	99	63%	57	69%	ns
Past & current	84	35%	58	37%	26	31%	
Total	240	100%	157	100%	83	100%	
**Tobacco use**							
Never	188	78%	121	77%	67	81%	ns
Past & current	52	12%	36	23%	16	19%	
Total	240	100%	157	100%	83	100%	
**Flossing use**							
No	104	43%	74	47%	30	36%	ns
Yes	136	57%	83	53%	53	64%	
Total	240	100%	157	100%	83	100%	

Chi-squared tests used to determine differences between Mexican and Latino immigrants.

* = Mean ages were 35.47 years (SE 0.74) for Mexican born and 35.58 years (SE 1.10) for those born in other Latino countries.

† = Length of residence in U.S.A. for Mexican immigrants was 11.43 years (SE 0.68) and Latino immigrants was 9.00 years (SE 0.79).

‡ 36 missing.

Table 2 presents epidemiologic variables for the subjects by place of birth and OHSI scores. The bivariate analysis in this table shows that Mexican immigrants have a significantly higher oral health status compared to other Latino immigrants (p < .05). Most of these differences in dental caries experience between the two groups are accounted for by the higher number of Missing Teeth (MT, p < .05). There were also statistically significant differences (p < .05) in the percentage ratios of Replaced Teeth/Missing Teeth.

**Table 2:** Comparison of mean values for OHSI and epidemiologic variables for Mexican and other Latino immigrants

	**Means (SE)**			
**Epidemiological Indices**	**Total (n=240)**	**Mexican Immigrants (n=157)**	**Latino Immigrants (n=83)**	**p-values**
**OHSI Scores**	82.35 (1.03)	83.90 (1.20)*	79.30 (1.90)*	<.05
**TOOTH STATUS**				
DMFT *	13.14 (0.46)	12.48 (0.54)	14.53 (0.81)	<.05
DT	2.23 (0.17)	2.10 (0.19)	2.47 (0.34)	ns
MT	4.96 (0.28)	4.53 (0.33)*	5.77 (0.50)*	<.05
FT	6.01 (0.36)	5.86 (0.44)	6.29 (0.64)	ns
DFT	8.23 (0.33)	7.96 (0.41)	8.76 (0.58)	ns
% DT/ DFT	37.19 (2.51)	36.89 (3.08)	37.77 (4.37)	ns
No. Sound Teeth	18.80 (0.46)	19.52 (0.54)*	17.47 (0.81)*	<.05
% Replaced Teeth /MT	6.03 (0.89)	4.49 (0.93)*	8.78 (1.63)*	<.05
**ATTACHMENT LOSS (AL)**				
mm of AL / person	1.95 (0.06)	1.94 (0.07)	1.97 (0.11)	ns

*t*-test used for comparisons.

* Scores based on 32 teeth.

ns = Not significant at p < .05.

Table 3 presents a multiple linear regression analysis, using the OHSI as the dependent variable and Mexicans and Other Latinos as the independent variable. This analysis presents epidemiologic, demographic and behavioral variables. All of the epidemiologic variables were highly significant (p <0.001) with mm of attachment loss at the mesial site having the largest coefficient (−9.841). In the analysis of the demographic variables, p < 0.01 level, indicating that other Latinos’ OHSI scores are five points lower than that of the Mexicans. As one would expect, covariate age, a continuous variable, was significant at the p < 0.05. Neither education nor income was significant and none of the behavioral variables (alcohol, tobacco and flossing) were significant. It is interesting to note that none of the behavioral variables were significant in explaining variation in the OHSI, even though other studies using perceived variables of oral health status, found education and tobacco to be significant. The gender differences between the two immigrant groups in our study were interesting. In general populations, females usually have better oral health than males [21,22]; however in this study, males had significantly better oral health than females. 

**Table 3:** Multiple linear regression analysis of the OHSI by epidemiologic, demographic and behavioral variables

**Independent variables**	**Regression coefficient**	**S.E.**	**p**
**Place of birth**			
Mexico	reference		
Other Latinos	- 5.048	1.615	<.01
**% DT/ DFT**	- 0.094	0.021	<.001
**No. RT/ MT**	- 3.097	0.707	<.001
**mm AL at Mesial**	- 9.841	0.885	<.001
**Age**	- 0.166	0.084	<.05
**Gender**			
Male	reference		
Female	+ 3.871	1.780	<.05
**Education**			
≤ Elementary School	reference		
Some High School or More	+ 1.737	0.634	ns
**Income**			
$0-$11,999	reference		
≥ $12,000	+ 0.870	0.580	ns
**Alcohol consumption**			
Yes	reference		
No	+ 1.741	1.697	ns
**Tobacco use**			
No	reference		
Yes	- 1.201	1.904	ns
**Flossing use**			
Yes	reference		
No	- 0.444	1.588	ns
**Beta Constant**	+126.278	5.750	<.001
Adj. R^2^ =	0.5124		

ns = not significant.

## Discussion

In an analysis of California residents, comparing whites, African American and Latinos, Hayes-Bautista *et al.* (1994) [23] characterized the Latino epidemiologic paradox in terms that defy the common wisdom that low-income, less educated groups, such as Mexicans, have low birth weights, high infant mortality, higher rates of heart disease, strokes and cancer, and other chronic diseases. His analysis showed that Mexican rates for these conditions were comparable to whites, even though their educational levels and incomes were lower, they did not access prenatal care and other health care and poverty rates were higher. This study presents a new perspective on the epidemiologic paradox by examining the relative oral health of Mexican and other Latino immigrants. In this study, all things being equal, Latinos who emigrated from countries other than Mexico have a lower OHIS index score compared to Mexican immigrants, indicating poorer oral health.

Education and income did not explain the differences in oral health status between Mexican immigrants and other Latino immigrants as indicated by the regression analysis. However, the epidemiologic paradox appears to exist, indicating that the Mexican immigrant lifestyle tends to promote significantly better oral health than other Latino immigrants. This occurred even though the Latino immigrants from other Latin American countries were exposed to more dentistry than the Mexicans, suggested by the higher number of filled teeth and the higher proportion of missing teeth that were replaced in this population.

Although the subjects from other Latino immigrant groups had more exposure to rehabilitative dental services, this did not result in better oral health according to the OHSI. This was primarily due to the effects of missing teeth on oral health because function and replacement of missing teeth do not restore oral health to the same extent as absence of disease. Therefore, the fact that Latinos who did not emigrate from Mexico had more exposure to dental treatment was not reflected in higher oral health status.

Similar to the study findings of Hayes-Bautista *et al.*[23], notably that while Mexicans had less prenatal care compared to whites their outcomes were comparable, our case suggests that Mexicans tend to have better oral health than other Latino immigrant groups while receiving less care. This may be due to the nature of the Mexican diet and high familial social support, which may reinforce good oral health practices. Another factor may be their reliance on community health fairs, which promote preventive practices. This is due in part to the fact that Mexican communities have existed in Los Angeles for a long time, enabling them to establish networks of health promotion even though access to care is a barrier for this population.

## Conclusions

Although this study is limited to a community-based sample, which thereby limits its interpretation and generalization, the results are important because they provide insight into populations that have been understudied. The study also demonstrates that place of birth (i.e., country/region of origin) is a significant factor in bringing to light differences in oral health outcome measures between Mexican immigrants and other Latino immigrant groups. Additionally, this study reinforces the concept of epidemiologic paradox within the Latino population; however, better models are clearly needed to capture more subtle differences within and between ethnic groups.

## Abbreviations

AL: Attachment Loss; ANOVA: Analysis of Variance between groups; DMFT Decayed: Missing, Filled permanent Teeth; DT: Decayed Teeth; DFT: Decayed Filled Teeth; FT: Filled teeth; Mm: millimeters; MT: Missing Teeth; NIDCR: National Institute of Dental and Craniofacial Research; OHSI: Oral Health Status Index; RT: Replaced Teeth; SD: Standard deviation; SE: Standard Error.

## Competing interests

The authors declare that they have no competing interests.

## Authors’ contributions

VWS designed the recording instruments, trained the backup examiner, conducted the dental examination, directed the data analysis and drafted the manuscript. MM was the PI on the center grant that included this survey, conceived the idea of conducting the survey and critiqued the manuscript. CDM conducted the data analysis, assisted in the interpretation of the analysis and assisted in the drafting of the manuscript. IDC was responsible for relating the conceptual model of health behavior to the demographic and behavioral variables. CAM was instrumental in revising the manuscript. All authors read and approved the final manuscript.

## Authors’ information

VWS is an epidemiologist with experience in large national surveys and in conducting clinical trials of preventive agents. MM is a health service researcher who studies oral health status. CDM is a sociologist who conducts research on ethnicity, immigrant populations and oral health. IDC is a health sociologist who has conducted research on complementary and alternative medicine and oral health quality of life. CAM is a medical anthropologist who has conducted community-based research in trauma, chronic disease, and oral health.

## Pre-publication history

The pre-publication history for this paper can be accessed here:

http://www.biomedcentral.com/1472-6831/12/39/prepub
